# Clinical comparative study assessing the effect of ivabradine on neopterin and NT-Pro BNP against standard treatment in chronic heart failure patients

**DOI:** 10.1007/s00228-022-03290-6

**Published:** 2022-03-03

**Authors:** Gaidaa M. Dogheim, Ibtsam Khairat, Gamal A. Omran, Sahar M. El-Haggar, Ahmed M. El Amrawy, Rehab H. Werida

**Affiliations:** 1 Pharmacy Practice Department, Faculty of Pharmacy, Alexandria University, Al Mesallah Sharq, Qism Bab Sharqi, Alexandria, Alexandria Governorate Egypt; 2 Cardiology Department, Faculty of Medicine, Tanta University, Tanta, Egypt; 3 Biochemistry Department, Faculty of Pharmacy, Damanhour University, Damanhour, 22514 Egypt; 4 Clinical Pharmacy Department, Faculty of Pharmacy, Tanta University, Tanta, Egypt; 5 Cardiology Department, Faculty of Medicine, Alexandria University, Alexandria, Egypt; 6 Clinical Pharmacy & Pharmacy Practice, Faculty of Pharmacy, Damanhour University, Damanhour, 22514 Egypt

**Keywords:** Ivabradine, Heart failure, Natriuretic peptide, N-Terminal-pro BNP, Neopterin

## Abstract

**Purpose:**

Heart rate reduction (HR) is a cornerstone in heart failure therapy as it improves patient outcomes. The aim of this study is to evaluate short-term effect of ivabradine on NT-Pro BNP and neopterin in heart failure patients and assess the association between HR and these biomarkers.

**Methods:**

Sixty patients on standard heart failure therapy were randomly allocated into ivabradine group (*n* = 30) and non-ivabradine group (*n* = 30). Ivabradine 5 mg twice daily was given for 3 months. Lipid profile and kidney functions were performed and blood samples for NT-Pro BNP and neopterin were analysed at baseline and after 3 months of intervention in both groups.

**Results:**

There was a significant improvement in NYHA class in ivabradine group (*p* < 0.001). Ejection fraction was improved in ivabradine and non-ivabradine groups after intervention (*p* < 0.001), with a greater improvement in ivabradine group (*p* = 0.026). Heart rate was reduced in ivabradine group (*p* < 0.001) and non-ivabradine group (*p* < 0.001) yet greater reduction was seen in ivabradine group (*p* < 0.001). Serum creatinine and blood urea nitrogen were reduced in ivabradine group (Scr: *p* = 0.001, BUN: *p* = 0.001). NT-Pro BNP and neopterin levels significantly decreased in ivabradine group (NT-Pro BNP: *p* < 0.001, neopterin *p* < 0.001). Significant positive correlation was found between HR and biomarker levels after intervention (NT-Pro BNP: *r* = 0.475, *p* < 0.001, neopterin: *r* = 0.384, *p* = 0.002).

**Conclusion:**

Ivabradine therapy reduced levels of both biomarkers which correlated well with HR. Biomarker levels might provide a tool for assessing ivabradine effectiveness in HF.

Trial registration

Date: June 26, 2020. Identifier: NCT04448899. Link: Ivabradine in Patients with Congestive Heart Failure—Full Text View—ClinicalTrials.gov.

## Introduction

Heart failure (HF) is a complicated clinical condition that affects the ability of the ventricles to fill up or pump enough blood to meet requirements of the body [1, 2]. HF incidence continues to increase in patients older than 65 [1, 2]. HF can be classified based on mechanism of dysfunction into either systolic (heart failure with reduced ejection fraction (HFrEF)) or diastolic heart failure (heart failure with preserved ejection fraction (HFpEF)) [1].

There are a variety of causes of HF, and the most common causes include: hypertension, ischemic heart disease (IHD) and diabetes [1–4]. Other causes of HF might include: cardiomyopathies, valvular heart disease, atrial fibrillation (AF), chronic kidney disease (CKD), chronic obstructive pulmonary disease (COPD), thyroid dysfunction and anaemia [1–4]. Hyperlipidemia has a major role in the progression of heart failure as demonstrated by previous studies. Hypercholesterolemia decreases coronary blood flow and induces apoptosis through diminished autophagy leading to LV dysfunction [5, 6]. Moreover, it promotes inflammation leading to tissue fibrosis. Studies found that high levels of fats lead to disrupting electrophysiology of the heart which in turn leads to arrhythmias [5, 6]. Recent findings found that levels of oxidised LDL are increased in HF leading to decrease in EF [5].

Symptoms may be due to either decreased cardiac output such as fatigue and weakness or can be related to fluid retention such as dyspnoea, paroxysmal nocturnal dyspnoea or wheezing [1]. Later, while the disease progresses, symptoms such as orthopnoea, anorexia, hepatic congestion, ascites and distended jugular vein are present [1]. Pulmonary congestion and lower extremity oedema are present in congestive HF pointing to the severity of the disease.

According to European Society of Cardiology (ESC) guidelines, pharmacological therapy for HF includes the use of angiotensin-converting enzyme inhibitors (ACEIs) or angiotensin receptor blocker/neprilysin inhibitor (ARNI) in addition to beta-blockers, mineralocorticoid antagonist (MRA) and dapagliflozin/empagliflozin as first-line therapy to reduce hospitalisation and mortality. In case of intolerance to ACEIs or ARNI, angiotensin receptor blockers (ARBS) are the alternative choice. Loop diuretics are used only to improve signs and symptoms of congestion with no effect on morbidity or mortality. Other agents include digoxin, hydralazine-isosorbide dinitrate (H-ISDN) and ivabradine [7, 8].

Ivabradine is an I_f_ channel inhibitor used for the treatment of HF that has been FDA approved in 2015 [9]. Ivabradine selectively and specifically inhibits I_f_ channel in sinus node which controls the spontaneous diastolic depolarisation and regulates HR resulting in a decrease in HR in patients with chronic HF [10, 11]. It has been FDA approved in patients with HFrEF who already are prescribed ACEIs/ARBs, beta-blockers and MRA and their sinus rhythm is ≥ 75 beats/min as an add-on therapy or as a replacement to beta-blockers [8]. Ivabradine is used in a dose of 5 mg twice daily. Target HR is between 50 and 60 beats/min [8].

Cardiac biomarkers have been related to the morbidity and mortality in HF patients. Example of those biomarkers is N-terminal-pro hormone brain natriuretic peptide (NT-Pro BNP). NT-Pro BNP is a 76-amino acid fragment resulting from the split of a pro-peptide into brain natriuretic peptide (BNP) and NT-Pro BNP [12]. NT-Pro BNP is inactive yet its level correlates better than BNP levels with clinical status of patients with HF [13, 14]. It has been proven that NT-Pro BNP levels are elevated in case of LV dysfunction and a prognostic marker for morbidity and mortality in HF. Persistently high levels of NT-Pro BNP predict poor outcomes [15, 16]. Neopterin is a 2-amino, 4-hydroxy pteridine compound which is a by-product of guanosine triphosphate bio-pterin pathway [17]. It is synthesised by active macrophages that are involved in inflammatory response of the immune system. It has a recognisable role in enhancing macrophage cytotoxicity through interaction with reactive oxygen, nitrogen and chlorides [18]. It is suggested to promote artherogenesis through oxidative stress-induced apoptosis in vascular smooth muscles and enhancing plaque growth [19, 20]. To date, neopterin levels are used in assessing the progression of viral infections, renal transplant rejection, nephritic syndrome and various autoimmune disorders [21]. Considering the pathophysiology of HF, immune system activation takes place and underlies the pathogenesis of the disease. Also, neopterin levels are closely related to progression of HF and there has been a relation between its concentration and state of HF [22].

It has been demonstrated that both levels of NT-Pro BNP and neopterin are elevated in patients with New York Heart Classification (NYHA) class II–IV HF [23–26]. Moreover, traditional therapies of HF demonstrated their ability to decrease those biomarkers as a part of their role to improve the patient’s condition. Thus, the use of both biomarkers may provide a novel promising tool to assess the efficacy of medications used in the management of chronic HF [13, 22, 27]. So, the primary objective of the present study was to evaluate short-term effects of ivabradine on NT-Pro BNP and neopterin. The secondary objective was to assess relationship between HR, NYHA and EF and both biomarkers.

## Materials and methods

### Study design and participants

A 3-month, double-blinded, parallel, interventional prospective randomised study was carried out on age, gender and BMI-matched adult patients. Sixty ambulatory, clinically stable symptomatic patients with systolic chronic HF (≥ 4 weeks), with left ventricular ejection fraction (LVEF) < 35%, NYHA class II–III and sinus rhythm and resting HR ≥ 70 beats/min on optimised standard medical therapy were included. Patients with acute decompensation, cerebrovascular events during the previous 6 months, pregnancy, breastfeeding, any valve dysfunction/abnormality, active myocarditis, second-degree and third-degree atrioventricular block and sick sinus syndrome were excluded from the study.

The included patients were recruited from out-patients’ clinics of cardiology department in Tanta University Hospital and Alexandria Main University Hospital (AMUH). The study took place from June 2020 to February 2021. All patients were evaluated clinically by expert cardiologists. Patients were randomly allocated using computer-generated random sequence in 1:1 ratio to enrol either in non-ivabradine group (*n* = 30) or ivabradine group (*n* = 30). All patients were on standard therapy of ramipril, bisoprolol, spironolactone, furosemide or atorvastatin at their maximum tolerated doses. Ivabradine group was on standard therapy in addition to ivabradine 5 mg twice daily, while non-ivabradine group received placebo twice daily. Doses of ivabradine were titrated upwards or downwards by 2.5-mg increments according to resting HR and tolerability. The study was done according to the Declaration of Helsinki. Approval was obtained from Research Ethics Committee of Faculty of Pharmacy, Damanhour University (No. 420PP25), and the trial was registered on clinicaltrial.gov by NCT04448899. All participants have agreed to be included in this clinical study and provided a written informed consent.

### Demographic data and baseline evaluation

Data on age, sex, weight, height, medical history and treatment at inclusion were collected. BMI was calculated through the formula: BMI = weight (kg)/ (height) ^2^ (m^2^). The baseline evaluation included physical examination, NYHA class, 12-lead electrocardiography (ECG) and ejection fraction (EF) by echocardiography. Laboratory tests including serum creatinine, blood urea nitrogen (BUN) and lipid profile and baseline levels of cardiac biomarkers NT-Pro BNP and neopterin were performed.

### Study procedures and biomarker measurements

An amount of 7 ml of venous blood sample was withdrawn from each patient between 8 and 9 am after a 30-min rest in the supine position into serum vacutainer test tubes, at the beginning of the study and after 3 months of intervention and follow-up. Blood samples were allowed to clot for 15–30 min then centrifuged at 3000 rpm for 15 min (Hettich Zentrifugen EBA 20, Tuttlingen, Germany). Serum was then divided into two portions: the first was used for direct determination of lipid profile (total cholesterol “TC”, triglycerides “TG” and high density lipoprotein “HDL-C”), while the second portion was frozen at − 80 °C until biochemical assay of biomarkers (NT-Pro BNP and neopterin) using commercially available ELISA kits (Sunred biological technology Co., Ltd., Shanghai). Fasting lipid profile was measured using the available commercial kits. TC and TG were determined using enzymatic colorimetric method (Allain et al. 1974; Bucolo and David 1973). HDL-C was determined using precipitation method. Low-density lipoprotein cholesterol (LDL-C) was calculated according to the Friedewald formula: LDL-C = [TC − HDL-C − (TGs/5)] provided that TG level is lower than 400 mg/dl [28]. All enrolled subjects were followed up during the study through clinical visits after 1 week of ivabradine administration and then at monthly intervals to assure response, compliance and reporting of any adverse effects towards the study medications.

### Statistical analysis

The collected data were analysed using software statistical computer package SPSS version 25.0 (SPSS Inc., Chicago, IL, USA). Continuous variables were expressed as means ± SD. Normality test was performed to ensure that data are normally distributed. Paired *t*-test was used to determine the difference within group between baseline and 3 months after treatment. Unpaired *t*-test was used to determine the difference between the two groups. Categorical variables are presented as number and percentage and analysed using the chi-square test. Wilcoxon signed-rank test was used to determine the difference between baseline and 3 months after treatment, while Mann–Whitney *U* test was used to determine the difference between the two groups for non-parametric variables. Safety and tolerability of ivabradine were assessed using relative risk (RR). Correlation between variables was evaluated using Pearson’s correlation coefficient. Area under receiver-operating characteristics (ROC) curve was used to evaluate the sensitivity and specificity of measured variables in HF patients. The significance level was set at *p* < 0.05. Post hoc sample size was calculated using G*Power software version 3.1.0 (Institut fur Experimentelle Psychologie, Heinrich Heine Universitat, Dusseldorf, Germany). It was estimated that total sample size of 60 patients would have a power of 98% to detect a medium to large effect size of 1.09 in the primary outcome measure.

## Results

### Subjects’ selection, randomisation and follow-up

**Fig. 1:**
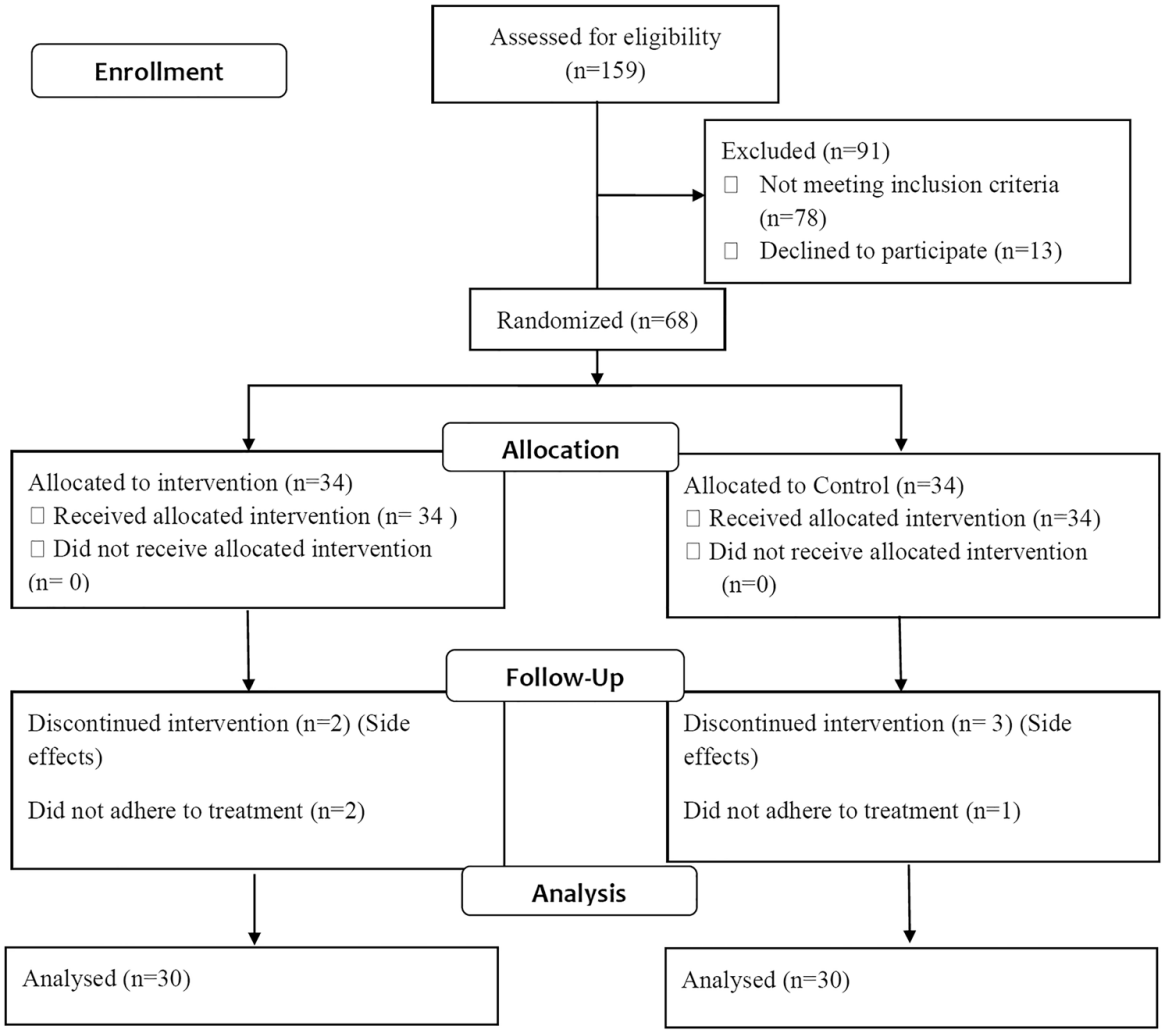
Consort flow diagram for participants’ screening, randomisation, allocation and follow-up

Screening, selection, randomisation and follow-up procedures of the study participants are illustrated in Fig. 1. Out of a total of 159 encounters, 78 did not meet the inclusion criteria and 13 declined to participate. Sixty-eight patients were enrolled and randomly allocated into two groups: ivabradine group (*n* = 34) and non-ivabradine group (*n* = 34). During follow-up, in ivabradine group, 2 patients did not adhere to treatment regimen and 2 discontinued the treatment due to incidence of acute HF. In non-ivabradine groups, 1 patient did not adhere to treatment regimen and 3 discontinued the treatment due to incidence of acute HF. Final analysis included 30 patients in ivabradine group and non-ivabradine group.

### Baseline characteristics of included patients

Baseline characteristics of included patients are shown in Table 1. Mean age of participants in ivabradine group (*n* = 30) was 55.63 ± 10.05 years old and in non-ivabradine group (*n* = 30) was 59.6 ± 10.0. Mean body mass index (BMI) was 29.08 ± 4.41 kg/m^2^ in ivabradine group and 28.99 ± 4.96 in non-ivabradine group. Most participants were males with a percentage of 73.33% in ivabradine group and 76.67% in non-ivabradine group. Mean HR was 83.7 ± 5.19 beats/min in ivabradine group and 84.27 ± 6.62 beats/min in non-ivabradine group, while mean ejection fraction was 25.6 ± 4.34% in ivabradine group and 25.77 ± 4.38% in non-ivabradine group. Most common medical history recorded for the participants was IHD (93.33%), HTN (60.0%) and DM (40.0%) in ivabradine group and IHD (86.67%), HTN (66.7%) and DM (50.0%) in non-ivabradine group. Dyslipidemia occurred in 33.33% in ivabradine group and in 36.67% of participants in non-ivabradine group. Commonly co-administered medications were ACEIs/ARBs (93.33%), beta-blockers (93.33%), spironolactone (83.33%), furosemide (70.0%) and statins (93.33%) in ivabradine group and ACEIs/ARBs (96.67%), beta-blockers (96.67%), spironolactone (76.67%), furosemide (73.33%) and statins (90.0%) in non-ivabradine group. There was no statistically significant difference in demographic data, medical history, laboratory tests or medication history in both groups (Table 1).

**Table 1:** Baseline characteristics of included patients

	Non-ivabradine, *n* = 30	Ivabradine, *n* = 30	*p*-value
Age (years)	59.6 ± 10.0	55.63 ± 10.05	0.131
Male	23 (76.67)	22 (73.33)	0.766
Female	7 (23.33)	8 (26.67)	
Weight (kg)	88.23 ± 13.77	86.33 ± 13.29	0.589
Height (cm)	174.87 ± 10.27	172.5 ± 8.67	0.339
BMI (kg/m^2^)	28.99 ± 4.96	29.08 ± 4.41	0.946
NYHA class II	8 (26.7)	7 (23.0)	0.559
NYHA class III	22 (73.3)	23 (76.7)	
Heart rate (bpm)	84.27 ± 6.62	83.7 ± 5.19	0.713
Ejection fraction (%)	25.77 ± 4.38	25.6 ± 4.34	0.883
Scr (mg/dl)	0.89 ± 0.18	0.98 ± 0.26	0.098
BUN (mg/dl)	22.87 ± 10.61	25.10 ± 12.21	0.452
LDL-C (mg/dl)	108.60 ± 33.97	106.83 ± 31.70	0.836
HDL (mg/dl)	42.47 ± 6.53	42.2 ± 7.17	0.881
Total cholesterol (mg/dl)	204.1 ± 27.19	199.4 ± 24.83	0.487
TGs (mg/dl)	134.23 ± 29.90	125.87 ± 31.54	0.296
Diabetes mellitus	15 (50.0)	12 (40.0)	0.436
Hypertension	20 (66.7)	18 (60.0)	0.592
Dyslipidemia	11(36.67)	10 (33.33)	0.787
IHD	26 (86.67)	28 (93.33)	0.389
Furosemide	22 (73.33)	21(70.0)	0.774
Spironolactone	23 (76.67)	25 (83.33)	0.519
Beta-blocker	29 (96.67)	28 (93.33)	0.554
ACEIs/ARBs	29 (96.67)	28 (93.33)	0.554
Statin	27 (90.0)	28 (93.33)	0.640

Data is represented as mean ± SD, numbers (percentages)

*NYHA* New York Heart Association, *Scr* serum creatinine, *BUN* blood urea nitrogen, *LDL-C* low-density lipoprotein C, *HDL* high-density protein, *TGs* triglycerides, *IHD* ischemic heart disease, *ACEIs* angiotensin-converting enzyme inhibitors, *ARBs* angiotensin receptor blockers

Unpaired *t*-test or chi-square as appropriate statistically significant between groups at *p* < 0.05

### Effects of ivabradine on NYHA classification, HR and EF

Table 2 shows the study outcomes for both groups at baseline and after 3-month follow-up. Non-ivabradine group showed no significant change in NYHA class compared to baseline. On the other hand, in comparison to baseline, ivabradine group showed a significant decrease in NYHA class (*p* < 0.001). Comparison between the two groups after 3 months of intervention shows significant decrease in NYHA class in ivabradine group (*p* = 0.002). There was a significant decrease in HR in both non-ivabradine group (*p* < 0.001) and ivabradine group (*p* < 0.001) (Table 2). Comparison between the two groups after 3 months of intervention shows a larger HR reduction in ivabradine group (*p* < 0.001). Significant increase in EF was observed in both ivabradine and non-ivabradine groups (*p* < 0.001) with a better improvement in ivabradine group as compared to non-ivabradine group after 3 months of intervention (*p* = 0.026) (Table 2).

**Table 2:** The study outcomes for both groups at baseline and 3 months after intervention

	Non-ivabradine (*n* = 30)		*p*-value^‡^	Ivabradine (*n* = 30)		*p*-value^‡^	*p*-value-between groups*		
	Baseline	3 months after intervention		Baseline	3 months after intervention		Baseline		3 months after intervention
NYHA class I No. (%)	0	0		0	9 (30.0)			0.002^⁋^	
NYHA class II No. (%)	9 (30.0)	10 (33.33)	0.317^§^	7 (23.3)	11 (36.7)	< 0.001^§^	0.559		
NYHA class III No. (%)	21 (70.0)	20 (66.7)		23 (76.7)	10 (33.3)				
EF (%)	25.77 ± 4.38	27.57 ± 3.31	< 0.001	25.60 ± 4.34	29.77 ± 4.11	< 0.001	0.883	0.026	
HR (bpm)	84.27 ± 6.62	80.10 ± 5.57	< 0.001	83.70 ± 5.19	65.60 ± 5.04	< 0.001	0.713	< 0.001	
Scr (mg/dl)	0.89 ± 0.18	0.88 ± 0.13	0.861	0.98 ± 0.26	0.89 ± 0.15	0.001	0.098	0.785	
BUN (mg/dl)	22.87 ± 10.61	23.03 ± 9.84	0.714	25.10 ± 12.21	20.33 ± 5.17	0.001	0.452	0.188	
LDL-C (mg/dl)	108.60 ± 33.97	107.53 ± 30.00	0.367	106.83 ± 31.70	107.23 ± 31.49	0.614	0.836	0.970	
HDL (mg/dl)	42.47 ± 6.53	43.17 ± 6.52	0.180	42.20 ± 7.17	42.37 ± 7.31	0.745	0.881	0.656	
T. cholesterol (mg/dl)	204.1 ± 27.19	203.37 ± 25.51	0.402	199.4 ± 24.83	200.40 ± 27.74	0.782	0.487	0.668	
TGs (mg/dl)	134.23 ± 29.89	132.27 ± 29.26	0.241	125.87 ± 31.54	124.40 ± 29.64	0.271	0.296	0.305	
NT-Pro BNP (pg/ml)	1180.9 ± 421.02	1152.87 ± 353.44	0.496	1127.67 ± 400.25	728.33 ± 293.37	< 0.001	0.618	< 0.001	
Neopterin (nmol/l)	93.4 ± 18.90	91.2 ± 17.56	0.210	89.57 ± 15.34	74 ± 13.67	< 0.001	0.392	< 0.001	

Data is expressed as mean ± SD, median (range) or as number (percentages) as appropriate

*NYHA* New York Heart Association, *EF* ejection fraction, *HR* heart rate, *Scr* serum creatinine, *BUN* blood urea nitrogen, *LDL-C* low-density lipoprotein C, *HDL* high-density protein, *TGs* triglycerides, *NT-Pro BNP* N-terminal-pro brain natriuretic peptide

^‡^Paired sample *t*-test or ^§^Wilcoxon signed-rank test statistically within groups significant at *p* < 0.05

^*^Independent sample *t*-test or ^#^chi-square or ^⁋^Mann–Whitney *U* test statistically significant between groups at *p* < 0.05

### Effects of ivabradine on kidney functions

Serum creatinine (*p* = 0.001) and BUN (*p* = 0.001) were significantly decreased in ivabradine groups, while there were no significant changes in non-ivabradine group (Table 2). Comparison between the two groups after 3 months of intervention showed no significant difference in kidney functions (Table 2).

### Effects of ivabradine on lipid profile

Table 2 shows that there was no significant change in lipid profile in either ivabradine or non-ivabradine groups after 3 months of intervention. There was no significant difference in lipid profile between the two groups after 3 months of intervention.

### Effects of ivabradine on NT-Pro BNP and neopterin biomarkers

**Fig. 2:**
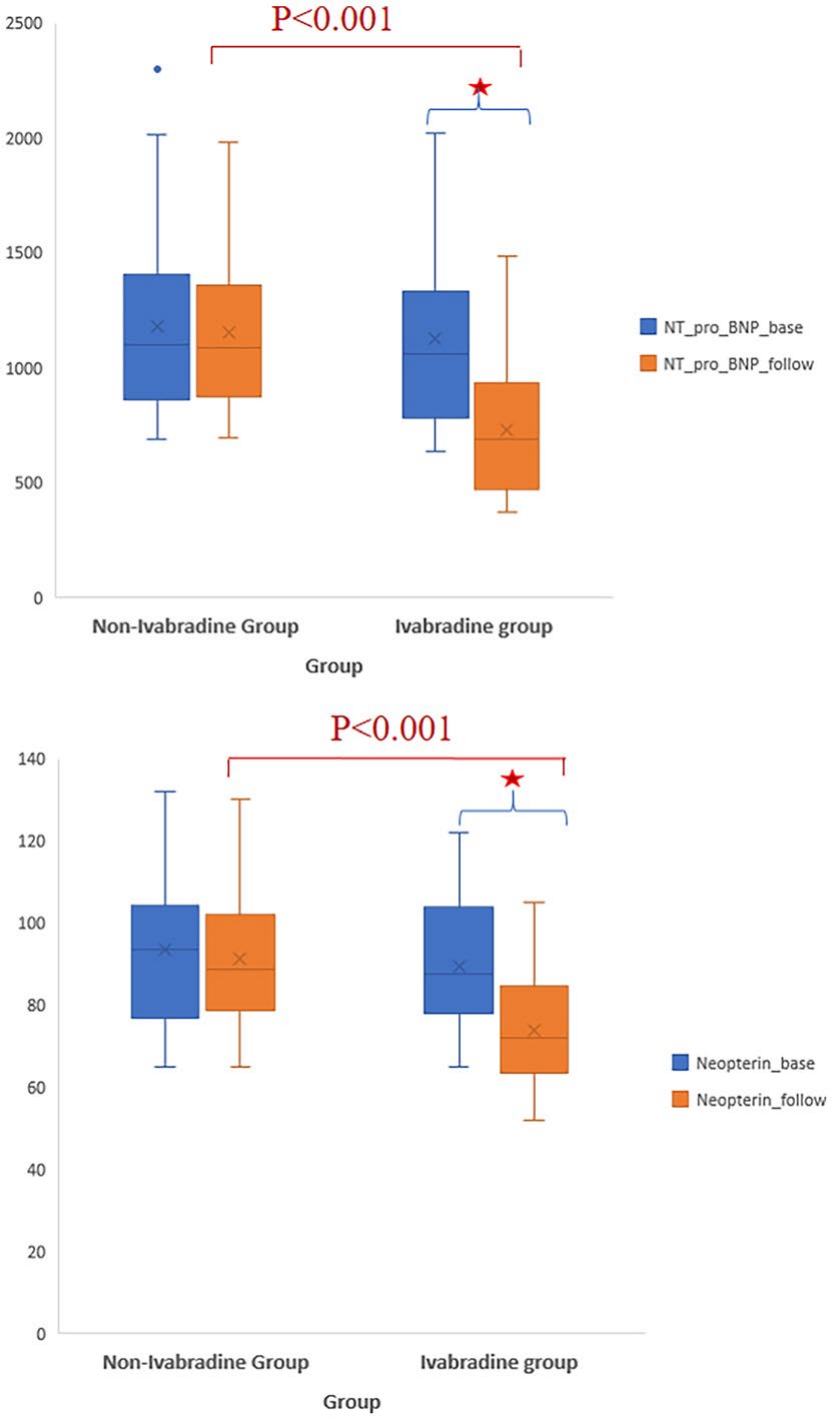
Change in NT-Pro-BNP and neopterin levels after intervention in the studied groups. NT-Pro BNP: N-terminal*-*pro brain natriuretic peptide. Statistically significant at *p* < 0.05

Table 2 demonstrates the effect of ivabradine on NT-Pro BNP and neopterin biomarkers at baseline and after 3-month intervention. There was a significant decrease in the levels of biomarkers NT-Pro BNP (*p* < 0.001) and neopterin (*p* < 0.001) in ivabradine group after 3 months of intervention as compared to baseline. On the other hand, there was no significant change in biomarker level in non-ivabradine group (Table 2). Comparison between the two groups after 3 months of treatment showed a significant difference between ivabradine group and non-ivabradine group in the levels of both biomarkers NT-Pro BNP (*p* < 0.001) and neopterin (*p* < 0.001) (Table 2) (Fig. 2).

### Correlation between NT-Pro BNP and neopterin biomarkers with NYHA class and ejection fraction in both groups

**Fig. 3:**
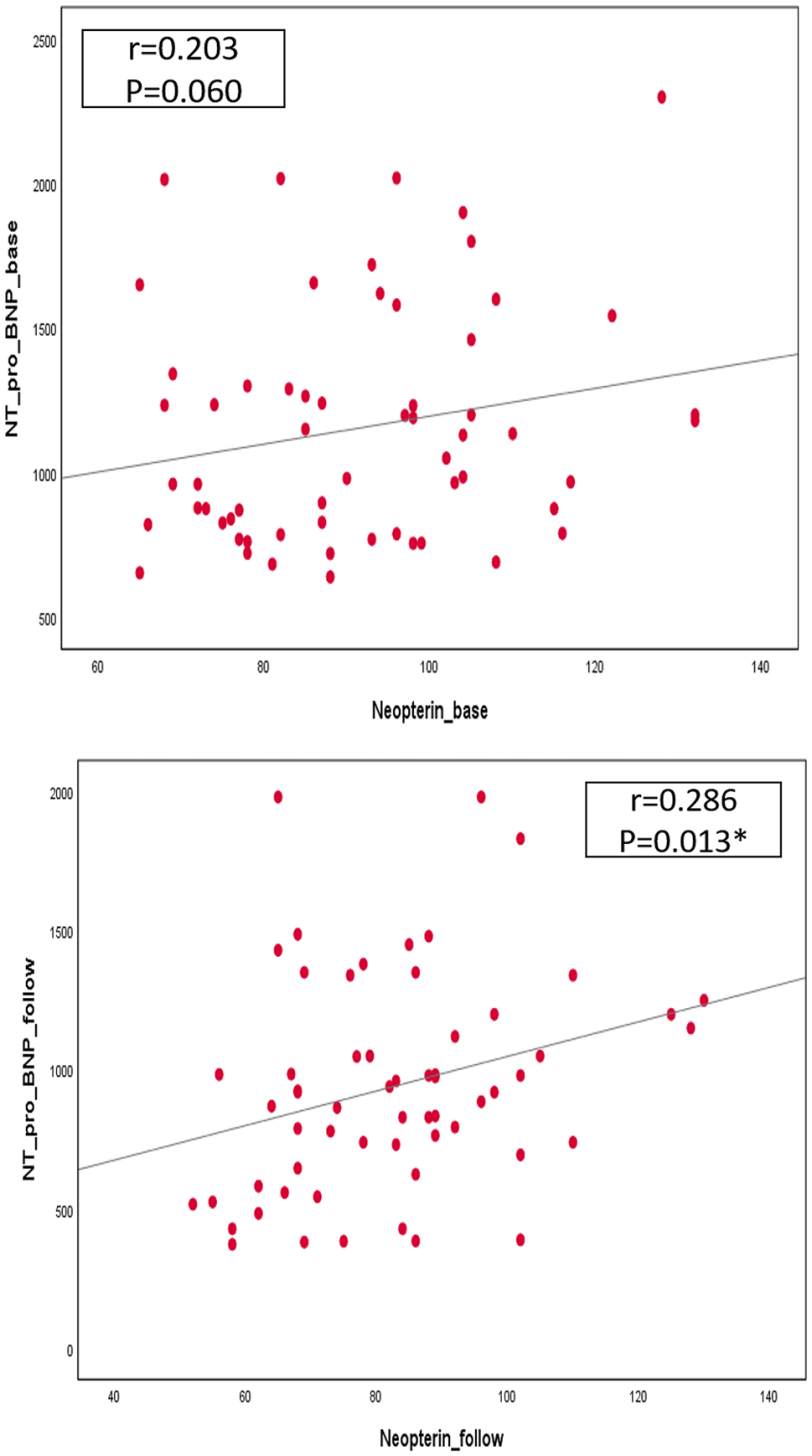
Pearson correlation of NT-Pro-BNP with neopterin in the studied groups before and after intervention. * NT-Pro BNP: N-terminal-pro brain natriuretic peptide*. *Statistically significant at p* < 0.05

Pearson’s correlation was carried out and illustrated in Table 3. There was no significant correlation between levels of biomarkers with NYHA class or ejection fraction. A significant positive correlation was found between NT-Pro BNP and neopterin with HR after intervention (NT-Pro BNP: *r* = 0.475, *p* < 0.001, neopterin: *r* = 0.384, *p* = 0.002). Also, a significant positive correlation was found between NT-Pro BNP with neopterin after intervention (*r* = 0.286, *p* = 0.013) as shown in Fig. 3.

**Table 3:** Pearson correlation between the measured parameters after intervention

	Baseline (*n* = 60)		After intervention (*n* = 60)		Baseline (*n* = 60)		After intervention (*n* = 60)	
	NT-Pro BNP		NT-Pro BNP		Neopterin		Neopterin	
	*r*	*p*	*r*	*p*	*r*	*p*	*r*	*p*
NYHA class	− 0.020	0.877	0.225	0.083	− 0.023	0.861	0.129	0.326
EF (%)	− 0.153	0.243	− 0.236	0.069	− 0.172	0.188	− 0.240	0.065
Heart rate (bpm)	0.087	0.509	0.475**	< 0.001	0.057	0.667	0.384**	0.002

*NYHA* New York Heart Association, *EF* ejection fraction

^**^Correlation is significant at the 0.01 level (2-tailed)

### Area under ROC curve of both biomarkers of the studied groups

**Fig. 4:**
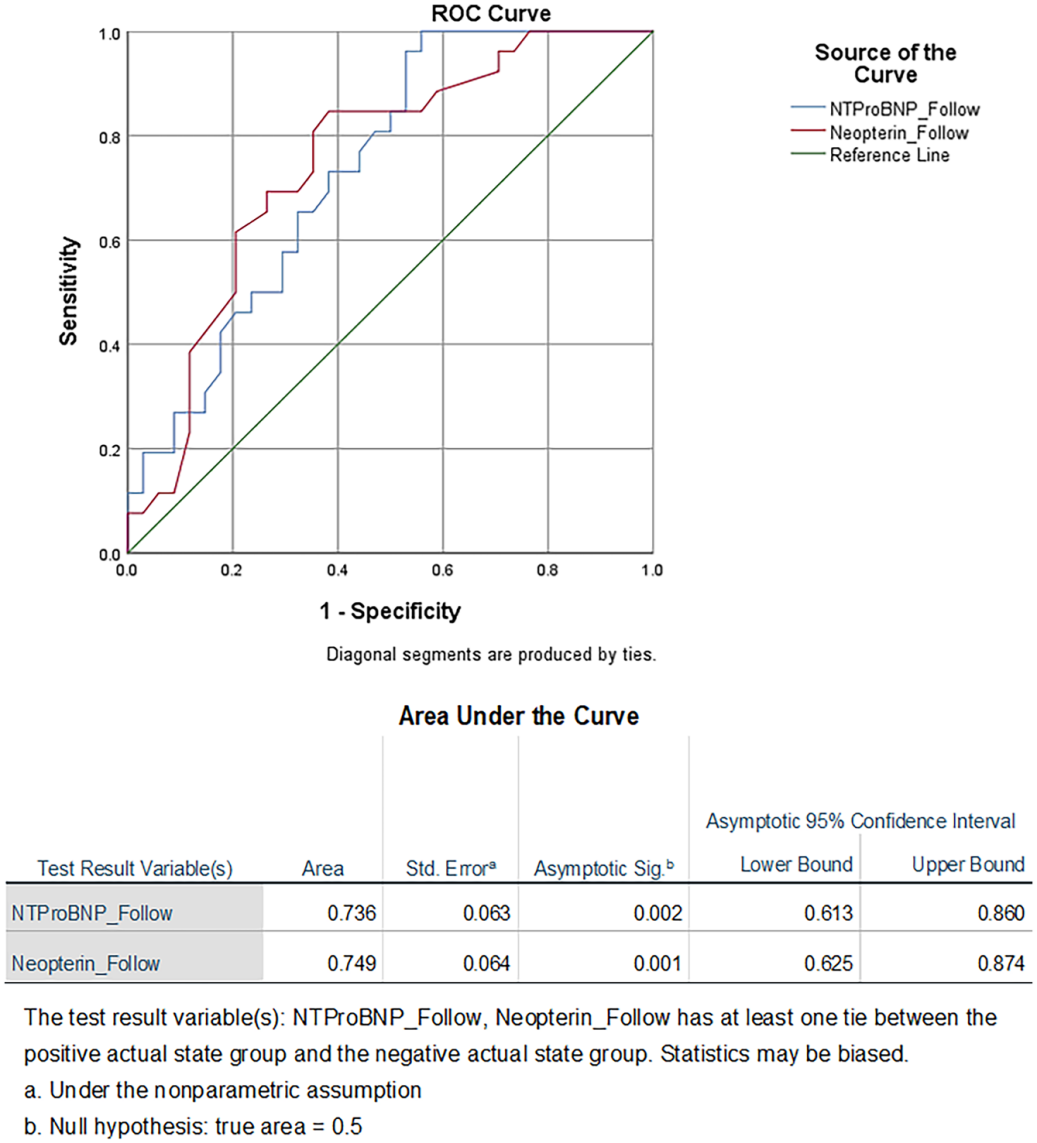
Area under ROC curve of both biomarkers of the studied groups. NT-Pro BNP: N-terminal-pro brain natriuretic peptide; Follow, follow-up

Figure 4 shows ROC-AUC of biomarkers in the treated groups. The AUC values associated with these ROC curves were 0.736 (*p* = 0.002; 95% CI 0.613–0.860) for NT-pro-BNP, and 0.749 (*p* = 0.001; 95% CI 0.625–0.874) for neopterin after 3 months of intervention.

### Reported adverse events in ivabradine group and non-ivabradine group

Table 4 illustrates the safety and tolerability of the studied medication. Out of 30 patients in each group completed the study, symptomatic bradycardia developed in 1 patient (3.33%) in ivabradine group and not developed in non-ivabradine group (*p* = 0.313). Asymptomatic bradycardia occurred in 2 patients in ivabradine group (6.67%) and 1 patient in non-ivabradine group (3.33%) (*p* = 0.554). Events of atrial fibrillation occurred only in 2 patients in ivabradine group (6.67%) (*p* = 0.150). Visual symptoms including phosphenes and blurred vision occurred in ivabradine group only (phosphenes: 1patient (3.33%), blurred vision: 1 patient (3.33%)) (*p* = 0.313).

**Table 4:** Reported adverse events for both studied groups

	Non-ivabradine (*n* = 30)	Ivabradine (*n* = 30)	RR	*p*-value
Symptomatic bradycardia	0 (0)	1 (3.33)	1.034 (95% CI: 0.968–1.106)	0.313
Asymptomatic bradycardia	1 (3.33)	2 (6.67)	2.071 (95% CI: 0.178–24.148)	0.554
AF	0 (0)	2 (6.67)	1.071 (95% CI: 0.974–1.179)	0.150
Phosphenes⁂	0 (0)	1 (3.33)	1.034 (95% CI: 0.968–1.106)	0.313
Blurred vision	0 (0)	1 (3.33)	1.034 (95% CI: 0.968–1.106)	0.313

Data represented as number (percentage)

*AF* atrial fibrillation, *HF* heart failure; ⁂transient enhanced brightness in a restricted area of visual field

*RR* relative risk for non-ivabradine/ivabradine

Chi-square significance level (*p* < 0.05)

## Discussion

The present study shows that in outpatients with chronic heart failure on optimised medical therapy and resting HR > 70 beats/min, the expected heart rate reduction with ivabradine therapy significantly decreases levels of NT-Pro BNP and neopterin after 3 months of intervention. Moreover, there was a direct relationship between HR reduction and both biomarker level reduction.

It has been well established that increased HR increases mortality and cardiovascular events in patients with HF. This risk markedly increases in patients with resting HR > 70 beats/min [29, 30]. Ivabradine selectively reduces HR through direct inhibition of the I_f_ current in the sino atrial node with no effect on myocardial contractility; thus, it preserves cardiac output and stroke volume [11, 31–33]. HR reduction attenuated left ventricular (LV) diastolic dysfunction, reduced left atrial (LA) and left ventricular structural remodelling and decreased LV collagen type I in hypercholesterolemic rabbits [34]. These effects can be seen as an improvement in EF after ivabradine therapy. Moreover, levels of angiotensin II and aldosterone were significantly decreased by ivabradine and correlated well with HR [34]. All these effects led to increased exercise tolerance and improved quality of life which are reflected as improvements in NYHA classification in patients receiving ivabradine therapy. The SHIFT trial demonstrated that ivabradine added to standard conventional therapy of HF led to an 18% decrease in the risk of cardiovascular death and hospitalisation for worsening HF [11].

NT-Pro BNP is a natriuretic peptide released in response to neurohormonal activation in patients with HF [24, 35]. Previous studies have demonstrated that ivabradine reduces the level of NT-Pro BNP in patients with HF. Moreover, Sargento et al. [36] demonstrated a positive correlation between HR and NT-Pro BNP level after intervention. Thus, NT-Pro BNP can be used in the diagnosis, prognosis and monitor the success of HF therapy [13, 14, 36–38].

Neopterin — a by-product of guanosine triphosphate bio-pterin pathway — is a marker of macrophage/monocyte activation. R. Caruso et al. established the relationship between neopterin levels and LV remodelling. They stated that high neopterin values correspond to abnormal LV diastolic dimension in HF patients. Thus, neopterin levels can be used as a prognostic tool to evaluate disease prognosis [39].

Lanser et al. demonstrated that neopterin levels correlate to disease severity and predict poor outcomes in HF patients. They also proved that neopterin is associated with LV dysfunction and increased cardiovascular risk which allow neopterin to be used as a marker of disease severity and prognosis [40].

Fuchs et al. and Ankrust et al. showed that neopterin levels increase in patients with HF. Moreover, as the disease progresses, levels of the biomarker increase remarkably; thus, neopterin can be used to confirm diagnosis and monitor disease progression [21, 41].

Wietlicka-Kokoszanek I et al. showed that neopterin levels are related to NYHA classification. Patients with NYHA class III had higher levels of neopterin than patients with NYHA class II [22].

Demir et al. demonstrated that neopterin is a powerful marker of inflammation in patients with HF. Thus, it can be used in the diagnosis of HF [23]. Moreover, they found that within 1-year follow-up, patients with high morbidity and mortality had higher levels of neopterin concentrations as compared to patients without risk of morbidity or mortality. One-year follow-up hospitalisation also correlated well with neopterin serum levels making it a valuable marker for disease prognosis [23].

In our study, neopterin levels were elevated in patients with HF in both groups at baseline. After 3 months of treatment with ivabradine, neopterin levels decreased in patients receiving ivabradine as compared to those receiving standard treatment. NYHA class and EF were improved in patients treated with ivabradine and HR decreased significantly after 3 months of intervention. There was no correlation between neopterin levels and either NYHA class or EF. Yet, results showed a positive relationship between the biomarker and HR. This can be explained by the fact that neopterin is released in response to myocardial distress and decline in cardiac function. Thus, as ivabradine decreases HR, reduces LV remodelling, improves LV filling and increases stroke volume resulting in improving cardiac function, biomarker level significantly decreases which makes it a possible candidate for monitoring effectiveness of ivabradine therapy in patients with HF [13, 14, 22, 36–38]. This study also demonstrated a positive relation between NT-Pro BNP and neopterin levels which is in agreement with Lanser et al. findings but contrary to R Caruso et al. which demonstrated no association between both biomarkers.

Regarding safety and tolerability of the studied medications, ivabradine has a favourable tolerability profile when used as monotherapy or in combination with beta-blockers [11]. The most common adverse reactions are bradycardia, atrial fibrillation and visual symptoms [10, 11, 42, 43]. Symptomatic and asymptomatic bradycardia are expected as adverse effects of ivabradine due to its direct inhibition of sino atrial node leading to lowering the HR [10, 11, 42, 43]. Yet, these adverse events occurred in a few patients ( asymptomatic bradycardia: 2 patients, symptomatic bradycardia: 1 patient) leading to only lowering the dose from 5 mg twice daily to 2.5 mg twice daily [10, 11, 42, 43]. Moreover, our results showed that the incidence of these adverse effects in ivabradine group is comparable to those in non-ivabradine group as reported in previous studies [44, 45]. However, more well-designed prospective trials are needed to investigate ivabradine’s additional impacts, particularly on adverse effects. Atrial fibrillation observed in this study has been reported in a number of previous studies [10, 11, 42]. In one study, ivabradine was seen to increase risk of AF by 15% which is higher than the percentage reported in product safety information [46]. AF as a side effect of ivabradine can be explained by the fact that ivabradine selectively inhibits HCN4 (hyperpolarisation-activated, cyclic nucleotide gated 4) channels which are the pore forming subunit of the funny channels in the SAN [47, 48]. This in turn slows sinus node diastolic depolarisation, while leaving sympathetic activation in the heart unopposed leading to induction of arrhythmogenesis which is masked by sinus rate reduction [49]. Regarding visual symptoms observed with ivabradine (phosphenes and blurred vision), this can be explained by the direct effect of ivabradine on the retinal ion channel (hyperpolarisation-activated cyclic nucleotide-gated channels (HCN)) which generates the I_h_ current [50, 51]. I_h_ current is the same family of the I_f_ current making it a target for inhibition by ivabradine. Luckily, incidence of visual symptoms is minimal and resolves spontaneously during or after treatment [10, 11, 42, 43].

### Study outcomes

To our best knowledge, this study is the first to assess the effect of ivabradine on neopterin biomarker. Levels of neopterin and NT-Pro BNP both decreased after 3 months of ivabradine therapy. This indicates that we may use both biomarkers to evaluate response of patients to ivabradine therapy.

### Study limitations

The sample size was relatively small which limits the generalisation of the results obtained from the study. Also, the intervention period was short. It is probable that longer follow-up might show more prominent changes in levels of both biomarkers. Thus, larger sample size studies for a longer period of time are required to better evaluate effect of ivabradine on biomarker levels allowing generalisation of results.

## Conclusion

While NT-Pro BNP has been demonstrated to be an excellent biomarker for chronic HF reflecting severity of the disease and effectiveness of therapy in a number of previous studies, the present study provides strong evidence that neopterin could be another biomarker of diagnosis of HF and improvement of EF and NYHA classification in patients treated with HF reducing drug ivabradine. More well-designed, larger-sample size, prospective trials are needed to investigate ivabradine’s additional impacts, particularly on end results.
